# Cortical Osteolytic Lesion in an Older Patient: Always Consider Metastasis

**DOI:** 10.5334/jbsr.3183

**Published:** 2023-05-31

**Authors:** Frederiek Laloo, Thiebault Saveyn, Wouter Huysse

**Affiliations:** 1 Ghent University Hospital, BE; 2 AZ Sint-Lucas Ghent, BE

**Keywords:** Adult, Femur, Bone Neoplasms, Lung Neoplasms, Neoplasms Metastasis, MRI

## Abstract

**Teaching point:** Cortical metastasis has to be considered in older patients with a cortical osteolytic lesion.

## Case History

A 53-year-old man with intense pain at the left upper leg was referred to a tertiary hospital for evaluation. The patient had already received magnetic resonance imaging (MRI) of the upper legs and pelvis. Radiography was not performed.

**Figure 1:**
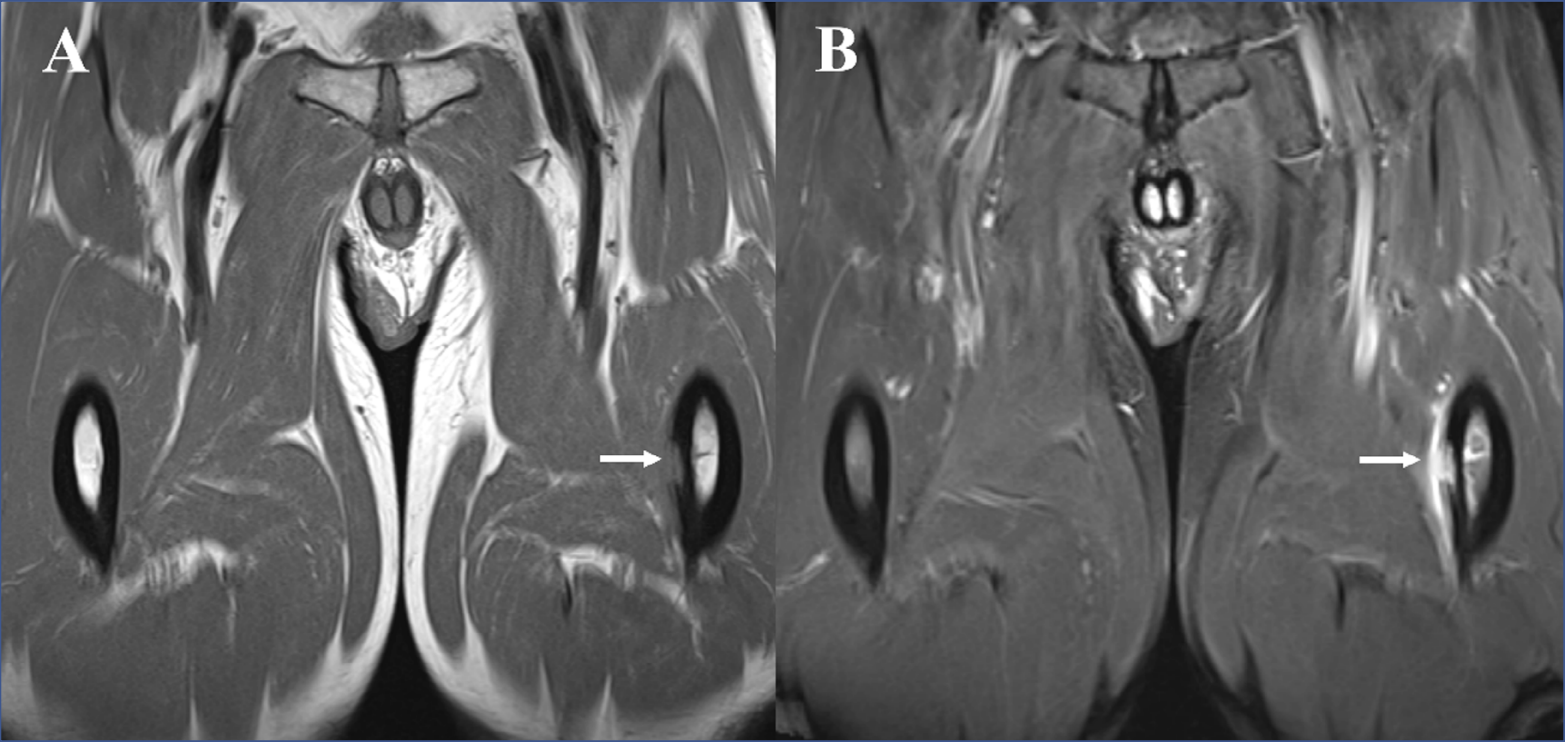


**Figure 2:**
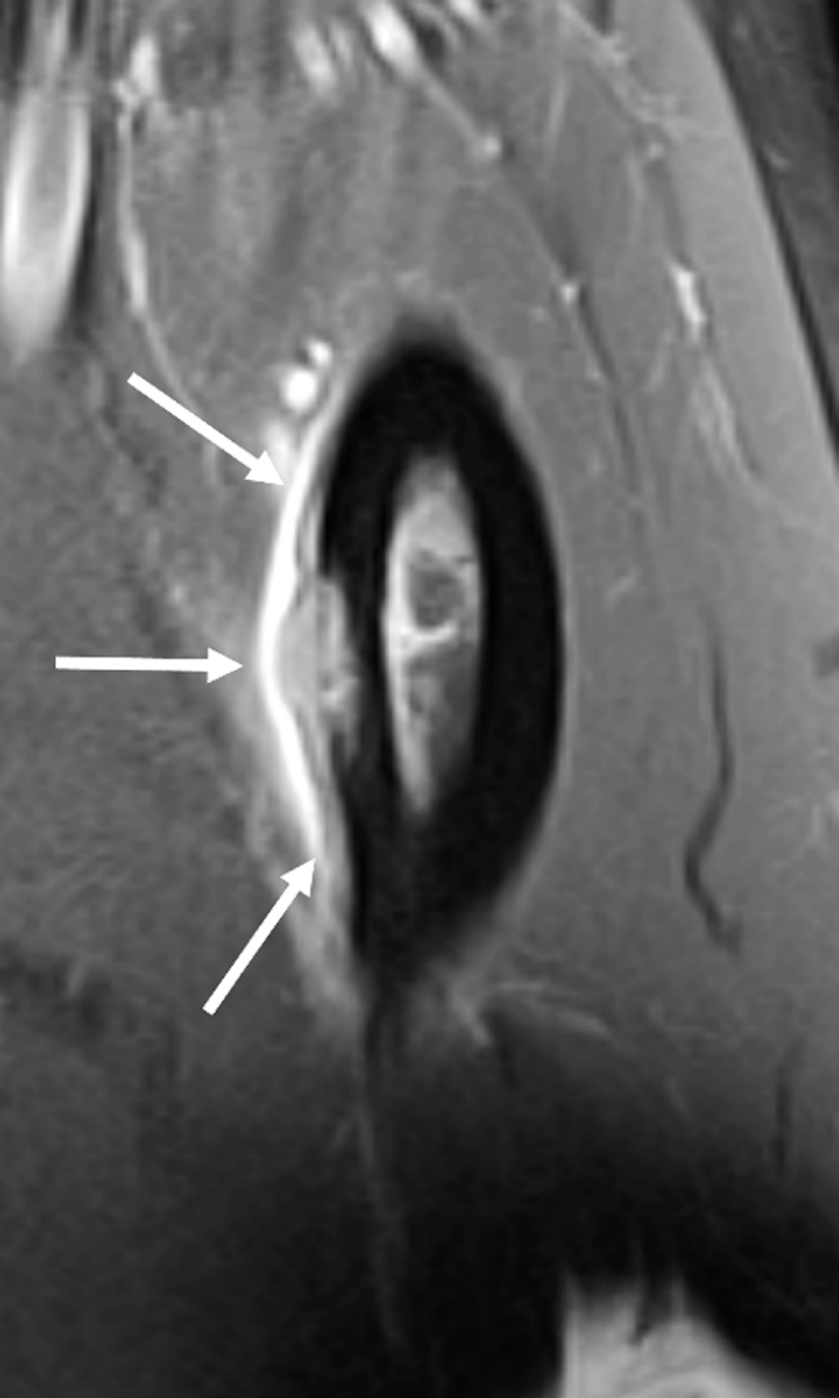


MRI showed a T1 isointense and T2 hyperintense lesion at the outer part of the medial cortex at the midshaft of the femur (Figure 1 A–B, arrow). There was no cortical thickening and only limited bone marrow edema. After intravenous administration of gadolinium, the precise location of the lesion could be seen as a moderately enhancing soft tissue mass with subperiosteal expansion and saucerization of the adjacent cortex (Figure 2, arrows). Avid enhancement of the periosteum was also present.

**Figure 3:**
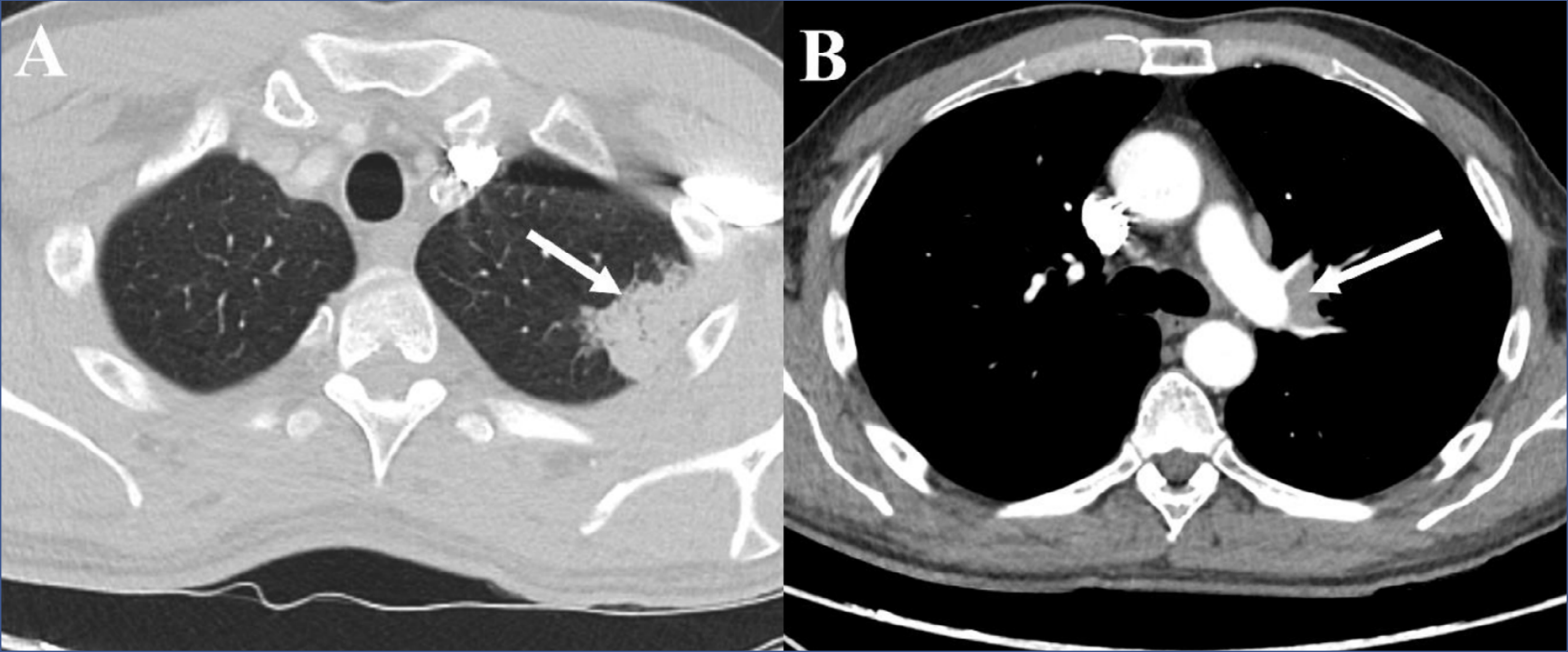


The primary diagnostic consideration was a cortical metastasis, and exploration on a primary tumor was initiated. The patient had a long-term history of smoking and computed tomography (CT) of the chest revealed a primary lung tumor (Figure 3A, arrow) with unilateral hilar lymphadenopathy (Figure 3B, arrow).

## Comments

Bone metastasis should be included in the differential diagnosis of multiple myeloma of an osteolytic lesions in patients of older age. When these two are excluded, the differential diagnosis can then be extended to other etiologies such as a Browns tumor in hyperparathyroidism.

Unlike the more typical medullary bone metastases—being more dependent on the formation of red bone marrow—cortical metastases are often seen at the level of the midshaft of the long tubular bones. They are hypothesized to originate from tumor emboli into the periosteal vascular network of the bones, which explains the extensive subperiosteal expansion as observed in this case.

Cortical metastases are typically from renal cell carcinoma or lung cancer (NSLCL), although they are also reported in primary tumors of the gastrointestinal tract, osteosarcoma, neuroblastoma, melanoma, hepatoma, breast carcinoma, and thyroid carcinoma [1].
